# Musculoskeletal surgeons use mixed reasoning rather than pure Bayesian strategies in clinical practice

**DOI:** 10.1371/journal.pone.0351694

**Published:** 2026-06-12

**Authors:** Robert Parisien, Alexander Drost, Amin Razi, Sina Ramtin, David Ring, Stein J. Janssen

**Affiliations:** 1 Elliot Orthopaedic Surgical Specialists, Elliot Hospital, Manchester, New Hampshire, United States of America; 2 Department of Surgery and Perioperative Care, Dell Medical School, Austin, Texas, United States of America; 3 Department of Orthopaedic Surgery, Amsterdam University Medical Center, University of Amsterdam, Amsterdam, Netherlands; Menoufia University, EGYPT

## Abstract

**Objectives:**

To inform efforts to promote regular and normalized Bayesian reasoning, we studied factors associated with the degree to which surgeons use Bayesian reasoning to navigate uncertainty across different clinical scenarios.

**Methods:**

Science of Variation Group members (153; 58% North America, 30% Europe, 69% over 15 years of experience) completed an online survey reading 8 scenarios of test and treatment decisions and chose one of 4 answer options with higher scores indicating more Bayesian reasoning. Internal consistency of the survey was assessed using Cronbach alpha.

**Results:**

The average Bayesian reasoning score across all scenarios was 3.0 (IQR 2.7–3.2) on a 4-point scale, indicating a relative context-dependent variability. Completely non-Bayesian reasoning was selected least often (8.6%, 90 of 1,044) and fully Bayesian reasoning represented 29% (301 of 1,044) of responses. Most surgeons showed mixed patterns (defined as reasoning in which prior probability is acknowledged but underweighted, without explicit probabilistic updating): 85% (121 of 142) used fully Bayesian reasoning at least once (121 of 142) while 42% (60 of 142) used completely non-Bayesian reasoning at least once. The Cronbach alpha was 0.43 suggesting the scenarios measured different aspects of clinical reasoning rather a unified construct.

**Conclusions:**

The finding that surgeons use relatively context-dependent reasoning suggests an opportunity for surgeons to develop and practice Bayesian reasoning strategies both in training programs and in practice.

## Introduction

### Background

Most medical decisions are made under conditions of uncertainty. Clinicians assess probabilities based on knowledge and evidence while navigating incomplete information and unresolvable uncertainty to advise people about the potential benefits and harms of visits, tests, and treatments. Bayesian reasoning provides a normative framework for this process, involving an initial probability estimate (the prior), systematic revision based on how strongly new evidence supports a given hypothesis (the likelihood), producing an updated probability (the posterior) [1]. This iterative, probabilistic process mirrors the dynamic nature of clinical decision-making, where surgeons integrate new evidence to revise probabilities based on prior knowledge such as clinical experience, research findings, and patient-specific factors to guide decisions.

Bayesian reasoning is particularly applicable in surgery where decisions often cannot be reversed, and both over- and under-treatment carry notable potential for harm. Despite growing emphasis on Bayesian principles in medicine and research [2,3], this framework remains under-emphasized in medical education and daily practice [4–6]. Research suggests clinicians adjust their diagnostic judgments as new evidence emerges but whether this reflects deliberate Bayesian reasoning or less systematic processes remains unclear [7,8].

Research in medical decision-making, psychology, and cognitive science consistently shows that while people understand the idea of updating beliefs with new evidence, they frequently struggle to do so accurately in practice [9,10]. This discrepancy arises because human cognition relies on mental shortcuts (heuristics) that can lead to systematic errors — such as base rate neglect, uncertainty intolerance, anchoring, and confirmation bias — which interfere with optimal probability updating [11–17]. Neutralizing these cognitive errors requires deliberate cultivation of critical thinking skills, which can be challenging in time-pressured clinical environments [18]. Despite the theoretical appeal of Bayesian reasoning and its alignment with many clinicians’ reasoning styles, the application of Bayesian principles in clinical practice is inconsistent [11,12,19–22].

### Rationale

These considerations raise important questions about how often and how effectively surgeons employ Bayesian reasoning in everyday practice [23,24]. The variability in how surgeons integrate evidence likely reflects multiple influences, including differences in clinical training environments, emphasis on critical thinking as opposed to unchecked assimilation of clinician habits and customs (”hidden curriculum”), and underlying cognitive dispositions that shape decision-making styles [25–28]. Some clinicians may have trained in settings that explicitly emphasize probabilistic reasoning and critical appraisal, while others developed their approach through more pattern-recognition models [29]. Individual comfort with probability, tolerance for ambiguity, and reliance on experiential “rules of thumb” can all affect how new information is incorporated [30–34].

Our goal in this study is to explore how musculoskeletal surgeons reason under common scenarios of uncertainty focusing on the spectrum of strategies, ranging from non-Bayesian approaches (threshold-based or heuristic) to fully Bayesian reasoning with explicit probabilistic updating. Rather than making value judgments, we aim to better understand how surgeons think in uncertain situations and to identify patterns that could inform future efforts to support effective clinical decision-making focused on quality and safety of care. Non-Bayesian reasoning patterns — particularly base rate neglect and over-reliance on objective test results — have been associated with tangible clinical harms, including unnecessary surgery, overdiagnosis, and failure to appropriately reassure patients in low-probability scenarios; understanding the prevalence and context-dependence of these patterns is therefore a prerequisite for designing interventions that reduce avoidable harm.

### Questions

As a next step towards these goals, we conducted a survey- and scenario-based experiment of musculoskeletal surgeons to evaluate the prevalence and variability of Bayesian reasoning in surgical practice asking: (1) Do musculoskeletal surgeons employ Bayesian reasoning in their clinical decision-making? (2) Is there variation in how surgeons utilize Bayesian reasoning across different clinical scenarios?

## Materials and methods

### Study design and setting

In a survey- and vignette-based experiment participants viewed eight scenarios a selected from among 4 response options testing Bayesian reasoning. The eight scenarios and options were developed by the lead author and edited and affirmed by the research team. The scenarios and options assessed how orthopaedic surgeons navigate clinical decision-making under common situations of uncertainty (Appendix 1). Specifically, the scenarios were designed to test specific aspects of Bayesian reasoning including probability updating, interpretation of test results, and integration of prior probabilities with new evidence. Each scenario had four possible responses reflecting different levels of Bayesian reasoning. Answers were ranked (1–4) according to degree of Bayesian reasoning and were listed in randomized order (Table 1). Firstly, to mitigate potential confounding, we iteratively pilot tested the scenarios by vetting the scenarios by a diverse group of local surgeons to ensure the clinical content was accessible to general musculoskeletal surgeons, regardless of sub-specialization. Secondly, the design was focused on structure over content by prioritizing the reasoning process rather than factual, clinical knowledge (e.g., updating priors based on new data). Thus, if a surgeon with a lesser knowledge of the specific pathology would still be able to answer appropriately by applying the required logic. Third and lastly, we interpreted the observed variation across scenarios not as an artifact, but as a key finding. The survey was distributed on December 2024 to members of the Science of Variation Group (SOVG). Two weekly reminders were provided and participation closed in January 2025. All participating surgeons treat musculoskeletal pathophysiology including orthopaedic surgeons, European trauma surgeons (who also treat fractures), and plastic hand and wrist surgeons.

**Table 1:** Ranked answer options according to Bayesian reasoning.

**ANSWER 1**	**Non-Bayesian Reasoning:**
	Prior probabilities not acknowledged
	Objectively based, deterministic reasoning
	Binary/absolute interpretation of evidence
**ANSWER 2**	**Mostly Non-Bayesian Reasoning with Evidence Emphasis:**
	Prior probability acknowledged but not incorporated
	Defaults to objective findings/testing
	Over-weights newer evidence or test results
**ANSWER 3**	**Mixed Reasoning with Contextual Integration:**
	Prior probability acknowledged but underweighted
	Lacking explicit probability updating
	Active consideration of context and priors
**ANSWER 4**	**Full Bayesian Reasoning:**
	Explicitly incorporates prior probability
	Systematic probability updating with new evidence
	Clear framework for evidence integration

### Ethical considerations

The study protocol was reviewed and approved by the Institutional Review Board. Completion of the survey was accepted as informed consent.

### Scoring validation

For construct validity, a normative Bayesian rubric was developed to score responses. Each response option was ranked (1–4) based on adherence to Bayesian principles: specifically, the correct application of prior probabilities (base rates), the use of likelihood ratios to update beliefs, and the avoidance of logical fallacies such as base-rate neglect or zero-risk bias. The detailed mathematical justification for each scenario’s scoring including priors, likelihoods, and posterior calculations is provided (Appendix 2).

### Participants/study subjects

This study utilized a convenience sample of musculoskeletal surgeons recruited through the SOVG. One hundred and fifty-three surgeons participated, with 124 complete responses on all 8 scenarios, and 142 participants who answered at least one scenario (in other words, 18 partial responses). Partial responses are usable in this study design. However, this approach assumes responses are missing at random; if non-completion is related to reasoning difficulty or discomfort with certain scenarios, this could introduce bias, though the direction of such bias is unclear. The respondents were predominantly from North America (58%) and Europe (30%). Most participants (69%) had extensive clinical experience and worked in academic practice (75%). Sixty-nine percent had over 15 years in practice (Table 2). For our purposes, it was sufficient to measure Bayesian reasoning in a relatively engaged and academic cohort, but this subset of surgeons (and arguably no subset) is representative of the average surgeon.

**Table 2:** Baseline characteristics of participants (N = 142).

		Percent	Number
**REGION**	North America	58	82
	Europe	30	43
	South America	7.0	10
	Australia	2.8	4
	Asia	1.4	2
	Africa	0.70	1
**TEACHING**	Yes	75	106
	No	25	36
**YEARS IN PRACTICE**	0-5	3.5	5
	5-10	9.9	14
	10-15	18	25
	15-25	32	46
	25+	37	52

### Variables, outcome measures, data sources, and bias

To calculate total scores for individual surgeons, their responses on each 4-point scale were averaged by dividing the total score by the number of questions answered, resulting in a final score on the same 1-to-4 scale with a higher score indicating more Bayesian reasoning. We included the results of 18 participants that answered fewer than 8 scenarios using the average score per scenario completed. To ensure transparency and reproducibility, the de-identified dataset underlying these findings is available as S1 Dataset.

### Statistical analysis

The degree to which the 8 survey items addressed similar aspects of reasoning (internal consistency) was assessed using Cronbach alpha. We chose to treat the outcome as continuous (linear) because we are analyzing a composite score (the average of 8 scenarios). We prioritized this approach for interpretability; summary statistics like means and medians are intuitive, whereas the coefficients from complex ordinal models (like cumulative link models) are difficult to interpret practically and robustness; since we are averaging across multiple scenarios, the resulting score distribution approximates a continuous variable, making linear summaries appropriate for describing the variation in reasoning. Overall score distributions were evaluated using the Shapiro-Wilk test for normality. As the data demonstrated a significant deviation from normal distribution (p < 0.01), descriptive statistics are reported as medians with interquartile ranges (IQR)*.* Non-parametric statistics were used for descriptive purposes given the non-normal distribution; linear regression was used separately to assess associations between surgeon characteristics and Bayesian reasoning score. All statistical analyses were performed using Python 3.10 (statsmodels package in Python). A two-tailed p value < 0.05 was considered significant. SOVG studies have varied participation that we cannot anticipate or alter. The statistical power comes from the number of observations, which is always very high, rather than the number of observers. Adequate power is determined by the ability to detect significant associations. We included any scenario that was rated even if the participant did not complete the entire survey, because each scenario and rating are independent from one another. Adequate statistical power considerations are assessed by the ability to measure significant associations.

## Results

### Do musculoskeletal surgeons employ Bayesian reasoning in their clinical decision-making?

The median Bayesian reasoning score across all scenarios was 3.0 (IQR 2.7–3.2) on a 4-point scale (equivalent to a total score of 24, IQR 22–26). Without an external comparator, we interpret this score to reflect context-dependent variability rather than adherence to a universal Bayesian reasoning framework. While Answer choice 1 (completely non-Bayesian reasoning) was selected least often (approx. 9%), and Answer choice 4 (fully Bayesian reasoning) represented 29% of all responses, most surgeons showed mixed patterns—85% used fully Bayesian reasoning at least once, while 42% used non-Bayesian reasoning at least once.

**Fig 1:**
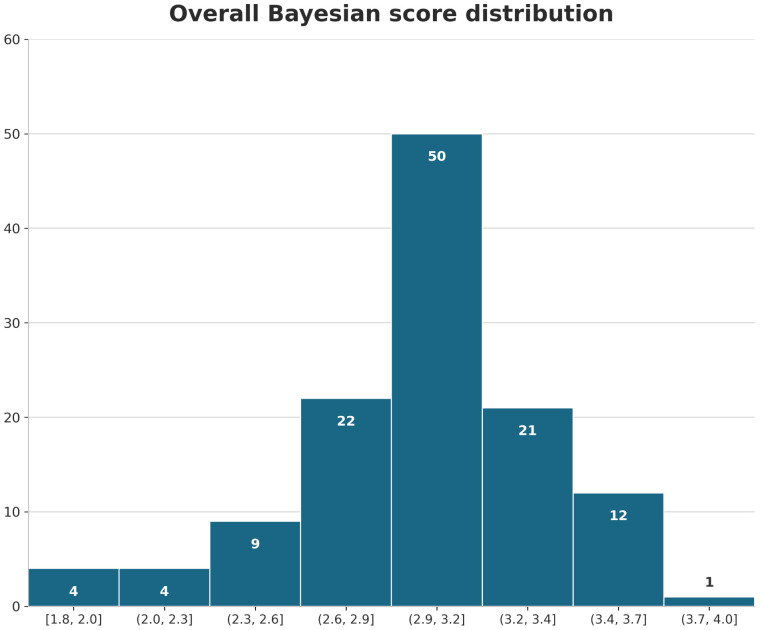
Overall Bayesian score distribution: A histogram of the overall Bayesian score per participant demonstrates 83% of scores (n = 118/142) between 2.5 and 3.5 with a negative skewness (−1.1) indicating more extreme low scores than extreme high scores. At the extremes, 6.5% scored in the non-Bayesian or mostly non-Bayesian range (1.8-2.3) while 12% demonstrated strong Bayesian reasoning (3.4-3.7).

The distribution of scores revealed a significant deviation from normality (Shapiro-Wilk *p* < 0.001), with 83% of scores clustering between 2.5 and 3.5 (Fig 1). The distribution showed a negative skew, indicating more extreme low scores (non-Bayesian) than extreme high scores. At the extremes, 6.5% scored in the non-Bayesian or mostly non-Bayesian range (1.8–2.3), while 12% demonstrated strong Bayesian reasoning (3.4–3.7). No participant selected fully Bayesian responses in all 8 scenarios.

### Is there variation in how surgeons utilize Bayesian reasoning across different clinical scenarios?

Internal Consistency analysis revealed a Cronbach alpha of 0.43, suggesting the scenarios measure somewhat different aspects of clinical reasoning rather than a unified construct. This scenario-specific variation was evident in the response patterns, ranging from predominantly mixed reasoning in some scenarios (e.g., Scenario 6: 60% choosing answer choice 3) to more Bayesian responses in others (e.g., Scenario 2: 50% choosing answer choice 4). The low Cronbach alpha indicates that how a surgeon reasons in one scenario does not strongly predict their approach in another, suggesting context-dependent adaptation of reasoning strategies rather than an overall disposition.

## Discussion

### Background, rationale, and general results

Clinical decision-making in orthopaedic surgery requires constant probability assessment and updating as new evidence emerges. While Bayesian reasoning provides a theoretical framework for this process, little is known about how surgeons address uncertainty in practice. This study examined how orthopaedic surgeons employ probabilistic reasoning in common clinical scenarios and found that surgeons generally use mixed reasoning strategies rather than consistently Bayesian or non-Bayesian approaches, with notable variation (both among individual surgeons and also collectively but without obvious patterns) across contexts. These findings are broadly consistent with prior investigations. Rottman and colleagues demonstrated context-dependent Bayesian updating in residents [7,8], and our results suggest a similar pattern persists among experienced attending surgeons. Our base rate neglect findings parallel those of Manrai and colleagues who found that physicians frequently miscalculate positive predictive value [9], and Teunis and colleagues who found only 11% of orthopaedic surgeons correctly applied base rate reasoning — a figure strikingly similar to our scaphoid scenario result [17]. Notably, our median score of 3 is higher than what purely non-Bayesian frameworks would predict, consistent with Croskerry’s adaptive expertise model suggesting that experienced clinicians develop context-sensitive rather than uniformly deficient reasoning strategies [27].

### Limitations

As with any survey-based experiment, a primary limitation is ecological validity. We can assume that surgeon responses in an online survey are not identical to approaches they may use with patients. The scenarios used in the survey necessarily simplified the complexities and dynamic nature of real-world decision-making. Nevertheless, the trends noticed are likely to reflect elements of variation in daily practice. And with additional influence of stress contagion, framing, and anchoring introduced by the referring clinician, the patient, and other aspects of the context, we anticipate greater variation and perhaps less Bayesian processing. Additionally, contemporary medical education emphasizes test-taking skills, which may lead surgeons to recognize and select a “normative” or preferred answer. Furthermore, some of the more Bayesian-oriented responses involved more complex probabilistic reasoning in the answer choice wording, which could further guide participants toward those choices. All experiments are subject to similar Hawthorne effects (the tendency of participants to modify their behavior when they know they are being studied) [35], but such effects do not seem to hinder reproducible and useful findings considering previous experiments conducted among members of the SOVG. A substantial limitation is that the SOVG sample — predominantly academic, highly experienced, and self-selected for engagement with research — is not representative of the average musculoskeletal surgeon. This non-representativeness likely operates in a specific direction: the SOVG cohort probably overestimates Bayesian reasoning relative to broader surgical populations, suggesting the true prevalence of non-Bayesian reasoning in everyday practice may be higher than our findings indicate. The finding of mixed reasoning in this relatively engaged and analytically inclined group may therefore represent an optimistic upper bound rather than a typical portrait of surgical cognition. Additionally, the skewed experience distribution — with only 3.5% of participants having fewer than 5 years in practice — may bias results toward reasoning patterns of senior surgeons and may not reflect the full spectrum of clinical reasoning across career stages.

### Do musculoskeletal surgeons employ Bayesian reasoning in their clinical decision-making?

The finding that surgeons of the SOVG employ context-dependent mixed reasoning strategies rather than consistently non-Bayesian or fully Bayesian approaches suggests that clinical reasoning in musculoskeletal surgery combines probabilistic thinking with other decision-making approaches based on clinical context. This integration of Bayesian and non-Bayesian approaches appears to reflect the practical realities of clinical decision-making, where pure probabilistic or pure non-probabilistic reasoning must be balanced against established protocols, clinical experience, and practical constraints. Our initial thought that surgeons might show a preference for non-Bayesian reasoning was only partially supported; while surgeons did not demonstrate strong Bayesian reasoning (which would require scores >3.5), they showed more sophisticated probabilistic thinking than expected. The distribution of responses, with most surgeons (85%) employing fully Bayesian reasoning in at least one scenario but few doing so consistently, suggests that surgeons possess the capability for Bayesian reasoning but apply it selectively rather than as a universal approach. Although linear regression identified slightly lower Bayesian reasoning scores among European participants (β = −0.23, 95% CI −0.41 to −0.055, p = 0.011), we interpret this finding cautiously given the small regional subgroup sizes, the non-representative nature of the SOVG sample, and the absence of data on specific educational or training system differences that might explain this variation.

It is important to distinguish between two possible explanations for mixed reasoning patterns. In some scenarios — most clearly the scaphoid scenario, where only 11% of surgeons correctly weighted a low prior probability against a positive CT — the non-Bayesian responses likely reflect genuine base rate neglect, a well-documented cognitive bias with direct clinical consequences. In other contexts, however, mixed or non-Bayesian responses may reflect adaptive expertise rather than cognitive failure. Croskerry (2018) argues that expert clinical reasoning is characterized not by uniform application of a single strategy, but by the ability to modulate reasoning approaches according to situational demands [27]. On this view, context-dependent reasoning is not synonymous with deficient reasoning. Our data do not allow us to fully distinguish between these two phenomena, and we acknowledge that both are likely present across the scenarios and surgeons studied.

### Is there variation in how surgeons utilize Bayesian reasoning across different clinical scenarios?

The finding of variation in how surgeons apply Bayesian reasoning across different clinical scenarios (low Cronbach alpha [0.43] and the distinct response patterns by scenario), suggests that heuristics, habits, norms, and emotions often override probabilistic reasoning in certain clinical contexts. Two interpretations of this low alpha are plausible and not mutually exclusive. First, the scenarios may not uniformly operationalize a single, unified Bayesian reasoning construct — they vary in structure, clinical domain, and the specific cognitive demands they place on the respondent, which could introduce measurement heterogeneity independent of true reasoning variability. Second, and consistent with our primary interpretation, Bayesian reasoning may not function as a stable individual trait but rather as a context-sensitive strategy that surgeons deploy selectively. The low alpha is compatible with both explanations, and we acknowledge that the instrument design does not allow us to fully distinguish between them. This ambiguity itself carries a meaningful implication: Bayesian reasoning may resist simple psychometric capture precisely because it is situationally expressed rather than dispositionally fixed [36].

For instance, in the twin MRI scenario (Scenario 2; in Table 3), 50% of surgeons demonstrated full Bayesian reasoning (answer choice 4), with an average score of 3.0. In contrast, the scaphoid scenario (Scenario 1; Table 4) had the lowest average score of 2.6, with only 11% of surgeons selecting the most Bayesian response. The scaphoid scenario may be more influenced by specific habits, norms, and emotions. Similarly, in the bone necrosis scenario (Scenario 4; Table 5), only about a third of participating surgeons maintained appropriate confidence in a high prior probability despite contradictory test results. In the delayed union scenario (Scenario 5; Table 6) also around a third of surgeons recognized that two experienced surgeons could legitimately arrive at different interpretations based on their prior experience with similar cases. These variations support the conclusion that surgeons may adapt their reasoning approach based on the specific clinical context rather than employing a consistent Bayesian or non-Bayesian strategy. This context-dependent reasoning is further supported by response patterns across scenarios. While most scenarios (particularly 1, 3, 4, 5, and 7) showed a predominance of mixed reasoning (answer choice 3), certain contexts appeared to trigger more Bayesian thinking. For instance, the twin MRI scenario’s clear contrast between prior probabilities and identical imaging findings made Bayesian reasoning more intuitive, prompting 50% of respondents to select the fully Bayesian option. Conversely, scenarios involving common clinical protocols or established norms, such as the scaphoid fracture scenario, tended to elicit more habitual or less probabilistic reasoning. These variations in reasoning highlight surgeons’ flexibility in adapting their approaches, which may explain the relatively low Cronbach alpha. It may underscore the adaptability of reasoning strategies to align with the unique demands of different scenarios. This adaptive variation is a hallmark of expert clinical decision-making, where surgeons must integrate probabilistic reasoning with heuristic and contextual approaches based on the specific challenges of each case.

**Table 3:** Scenario 2.

Two 45-year-old identical twins present with knee pain. Both have MRI scans where the radiologist describes their lateral meniscus findings in exactly the same way: “signal changes compatible with either degenerative changes or tear.” • Twin A is a competitive soccer player who developed pain and swelling after pivoting in a game three days ago. • Twin B works a sedentary desk job and describes worsening pain over several months, worse with stairs. Which interpretation do you most agree with?	Percent	Number
1. When MRI findings are truly identical, we must interpret and treat them the same way – introducing clinical factors risks bringing subjective bias into what should be an objective radiological diagnosis. *(Least Bayesian)*	1.5	2
2. While clinical histories differ, identical MRI findings usually indicate the same underlying process – we should start with similar treatment approaches and adjust based on response.	8.9	12
3. Despite identical imaging, clinical factors suggest we should modify our initial interpretation to account for their different situations and activity levels, though the underlying pathophysiology appears similar.	40	54
4. Despite identical imaging, the clinical context transforms our interpretation – the different prior probabilities (high for acute tear vs. low for degenerative changes) determine how we must interpret these identical MRI findings, leading to different diagnostic conclusions. *(Most Bayesian)*	50	67

**Table 4:** Scenario 1.

A patient presents with wrist pain after a fall. Before any testing, based on injury mechanism, symptoms and exam, you estimate the probability of scaphoid fracture is very low, around 5%. Their CT scan shows a possible fracture, and in your hospital CT scans for scaphoid fractures are 85% sensitive and 75% specific. When interpreting these results, which approach best matches your reasoning?	Percent	Number
1. I would trust the CT findings over clinical impression since evidence from objective imaging is more reliable than subjective assessment. *(Least Bayesian)*	11	16
2. I would consider both the CT and my clinical impression but rely more on the CT since it represents objective evidence.	24	34
3. I would integrate both the CT findings and my clinical impression, recognizing that a positive CT increases the probability but doesn’t make it certain.	54	76
4. Even with a positive CT, the probability of true fracture remains low (around 15%) because my initial estimate was very low. *(Most Bayesian)*	11	16

**Table 5:** Scenario 4.

A breakthrough new imaging technology is now FDA-approved after extensive validation that can detect bone necrosis in open fractures with very high accuracy. The test has been found to be 95% sensitive and 95% specific when tested in over 2000 patients. Before you order the test, you estimate that the probability of necrosis is nearly certain, about 99% due to clinical assessment. But to your surprise, the test comes back negative. What is the probability that the bone is necrotic?	Percent	Number
1. Unlikely – the accurate negative test essentially rules it out. *(Least Bayesian)*	4.6	6
2. Low probability – with a test this accurate, we should strongly favor an alternative diagnosis.	18	23
3. Moderate probability – this validated negative result requires us to carefully reconsider our diagnosis.	50	65
4. High probability – our initial near-certainty means it’s still probably necrotic. *(Most Bayesian)*	28	37

**Table 6:** Scenario 5.

A 67-year-old presents for a second opinion regarding a distal femur fracture treated with plate fixation 5 months ago. He has pain with weight bearing. Radiographs show a stable construct with some areas of persistent radiolucency at the fracture site, but without broken or loose implants. Two experienced trauma-fellowship trained surgeons evaluate the patient one week apart. • Surgeon A has managed many delayed unions and has observed that patients with stable constructs and improving function despite persistent pain and radiolucency often progress to union with continued protected weight bearing. Based on this experience, they recommend continuing current management with close monitoring. • Surgeon B has found in treating many similar patients that despite radiographs showing a stable construct and improved function, patients with persistent pain at this point have more predictable and faster outcomes with bone grafting. Which statement best represents your view on the nature of ‘truth’ in diagnostic interpretation?	Percent	Number
1. There must be an objective biological truth about the healing status of this fracture – additional imaging studies and biomechanical testing should definitively reveal whether adequate healing is present or not. *(Least Bayesian)*	9.4	12
2. While both surgeons’ experiences are important, we need standardized diagnostic criteria to determine which approach is correct in this setting.	18	23
3. Different practice contexts lead surgeons to develop different diagnostic frameworks – one might be closer to the truth, but both approaches could be valid paths to helping the patient.	37	47
4. The surgeons’ prior experiences with similar patients shape how they estimate probabilities of different diagnoses, leading to different but equally legitimate diagnostic interpretations. *(Most Bayesian)*	35	45

### Other relevant findings

#### Illustrative scenarios.

Key scenarios demonstrate distinct patterns in Bayesian reasoning application. In the scaphoid scenario (Scenario 1 as shown in Table 4), surgeons were given a positive CT scan in a low-probability situation (5% prior). Only 16/142 (11%) chose the most Bayesian response recognizing that even with positive findings, the posterior probability remains low (15%) due to the low prior probability. Instead, 35% of surgeons chose to trust the positive imaging over clinical assessment, demonstrating base rate neglect.

The twin MRI scenario (Scenario 2 as shown in Table 3) revealed surprisingly sophisticated Bayesian reasoning, with 50% of respondents demonstrating full Bayesian reasoning (answer 4) and only 1.5% selecting non-Bayesian options. However, this high rate of Bayesian responses may reflect scenario design limitations rather than typical reasoning patterns, as the identical MRI findings with contrasting clinical histories made the role of context unusually explicit.

In the bone necrosis scenario (Scenario 4 as shown in Table 5), only 28% of surgeons maintained appropriate confidence in a high prior probability (99%) despite contradictory test results. Most surgeons (72%) allowed a single negative test to override strong clinical suspicion, again demonstrating difficulty maintaining appropriate weighting of prior probabilities.

The delayed union scenario (Scenario 5 as shown in Table 6) revealed how clinicians sometimes struggle with probabilistic reasoning in practice. Only 35% of respondents accepted that different clinicians could legitimately arrive at different interpretations based on prior experience. This finding illuminates a broader challenge: the tension between probabilistic reasoning and the desire for definitive answers. Most clinicians favored seeking additional testing over acknowledging the validity of experience-based probability differences, suggesting discomfort with the inherent uncertainty in probabilistic reasoning.

#### Base rates and prior probabilities in clinical reasoning.

These results show evidence for base rate neglect in varying degrees in many surgeons’ responses to the scenarios. Base rates and prior probabilities form the foundation of Bayesian reasoning. The primary Bayesian insight is that test results gain meaning only when contextualized by prior probability. Base rate neglect is especially problematic because it directly undermines accurate probabilistic assessment. In clinical practice, the base rate might represent disease prevalence, complication likelihood, or the probability of specific injury patterns in given populations. Neglecting these base rates leads to flawed probability estimates, particularly when interpreting test results. For example, as demonstrated in Scenarios 1 and 4, even highly accurate tests can yield misleading interpretations if the base rate is very low or very high. Without considering base rates, clinicians might overestimate the significance of positive test results, leading to unnecessary interventions or missed alternative diagnoses [37].

The clinical consequences of base rate neglect can be quantified using the scaphoid scenario as a worked example. With a prior probability of 5% and a CT scan that is 85% sensitive and 75% specific, a positive result yields a posterior probability of approximately 15% — meaning the fracture remains unlikely despite the positive test. In a hypothetical cohort of 100 patients with this presentation, approximately 28 would receive a positive CT result, of whom roughly 24 would represent false positives. The 35% of surgeons in our study who chose to defer to the CT over clinical assessment would, in practice, expose the large majority of these patients to unnecessary immobilization, additional imaging, and the attendant costs and anxiety — all of which are avoidable through appropriate application of prior probability. This example illustrates that the gap between abstract reasoning scores and clinical outcomes is not merely theoretical: context-dependent base rate neglect has a direct and calculable impact on overtriage and overtreatment.

While priors can incorporate subjective clinical experience, the base rate provides an objective foundation for initial probability estimates. Previous research suggests surgeons struggle with this concept; Teunis et al. (2016) found only 11% of orthopaedic surgeons correctly answered questions requiring base rate consideration [17]. Our survey results demonstrated that fully considering base rates remains challenging, particularly when test results appear to contradict these fundamental probabilities.

#### Variation of reasoning in the era of AI.

This study should be understood as a variation‑of‑reasoning paper rather than a traditional variation‑of‑care analysis. Instead of examining differences in treatment patterns, we focus on the underlying cognitive processes that shape how surgeons interpret evidence and update diagnostic beliefs. The finding that surgeons employ context‑dependent, mixed reasoning strategies highlights meaningful heterogeneity in how clinicians navigate uncertainty, even when presented with identical information. This distinction is increasingly important in a moment when AI‑assisted clinical decision tools are poised to rely heavily on Bayesian or Bayesian‑like probabilistic updating, offering consistent, mathematically coherent reasoning. Our results suggest that human cognition does not always align with these normative probabilistic frameworks, raising timely questions about how surgeons will interact with AI systems, how discrepancies between human and machine reasoning may influence decisions, and how training might better prepare clinicians for a future in which Bayesian reasoning is embedded in the tools that support patient care [38].

## Conclusions

This study demonstrates that orthopaedic surgeons employ more sophisticated probabilistic reasoning than expected, though their approach is notably context-dependent rather than consistently Bayesian. These findings should be interpreted with the caveat that the SOVG sample represents a ceiling on external validity; results are likely not generalizable to the average practicing surgeon, and the true extent of non-Bayesian reasoning in broader surgical populations may be greater than observed here. While surgeons regularly incorporate elements of Bayesian thinking, they do so selectively based on clinical context, as evidenced by the median score of 3.0 and the low internal consistency across scenarios. The findings reveal specific challenges in clinical reasoning, particularly regarding base rate consideration and the acceptance of differing probability estimates based on prior experience. These insights suggest that future educational initiatives should focus not on rote adoption of Bayesian methods, but rather on helping surgeons recognize specific clinical contexts where probabilistic reasoning is most crucial for patient care. Case-based learning and scenario-specific training — rather than instruction in Bayesian formulas in the abstract — are likely to be most effective, as they mirror the contextual nature of the reasoning variability observed here and allow surgeons to practice probability updating within the kinds of clinical situations where base rate neglect is most consequential. Notably, years in practice was not associated with Bayesian reasoning score in this study (β = −0.017, p = 0.64), suggesting that clinical experience alone does not confer probabilistic reasoning proficiency — a finding with direct relevance for continuing medical education programs, which should not assume that senior clinicians are exempt from reasoning biases that formal training could address. Understanding how and when surgeons employ different reasoning strategies may ultimately lead to more nuanced approaches to clinical decision-making and more effective teaching of clinical reasoning in musculoskeletal surgery. Future research should explore how structured decision-making frameworks, and targeted educational interventions can optimize the application of Bayesian reasoning in musculoskeletal surgery. The context-dependence of reasoning observed here also has implications for clinical guideline implementation: guidelines that assume normative probabilistic reasoning as a uniform substrate may be applied inconsistently across surgeons and clinical contexts, suggesting that guideline design itself may need to account for the variability in how evidence is interpreted and weighted.

## Supporting information

S1 Dataset Deidentified dataset. This was the dataset used for the analysis of this study.
(CSV)
