# Supplementary material for: Musculoskeletal surgeons use mixed reasoning rather than pure Bayesian strategies in clinical practice
Source: PLoS One. 2026 Jun 12;21(6):e0351694. doi: 10.1371/journal.pone.0351694 (PMC13262866; doi:10.1371/journal.pone.0351694)
Supplement: Attachment — Submitted filename: Response to Reviewers.docx [file pone.0351694.s002.docx]

PONE-D-25-55463, Musculoskeletal Surgeons Use Mixed Reasoning Rather Than Pure Bayesian Strategies in Clinical Practice

**Response to Reviewers**

Dear Dr. Badr,

We appreciate the efforts of yourself and the editors to help improve our manuscript. We have addressed the comments in a revision as follows:

Reviewer #1:

1. Did you perform any test to determine the appropriate sample size?
Given the sensitivity of the study, please revise the manuscript to include complete details on sample size justification.

**Response: SOVG studies have varied participation that we cannot anticipate or alter. The statistical power comes from the number of observations, which is always very high, rather than the number of observers. Adequate power is determined by the ability to detect significant associations.**

**Changes: More explanation about statistical power is added to the manuscript.**

2. Please provide the full text of all eight scenarios, including the four response options for each scenario, as well as the detailed normative Bayesian justification (priors, likelihoods, posteriors, and the criteria used to rank options from non-Bayesian to fully Bayesian).
Only Scenarios 1, 2, 4, and 5 appear to be included; the full set of eight scenarios is not visible.

**Response/Changes: An appendix with all of the scenarios had already been added named “appendix 1”.**

3. In the section on “other relevant findings,” the references to Scenario 1 (Table 3) and Scenario 2 (Table 4) appear inaccurate.
Please review and correct the scenario numbering and corresponding table references.

**Response/ Changes: Scenario numbering has been corrected.**

4. How were the scenarios and their Bayesian rankings developed and validated?
Please indicate whether you conducted:
- Expert panel review
- Pilot testing
- Cognitive interviews
- Interrater agreement assessments for the Bayesian ranking criteria

**Response/Changes: Added a description of the development of the rankings based on knowledge and evidence, an expert panel, and experience in prior studies.**

5. Given that the primary outcome is ordinal, why did you choose linear models instead of ordinal or mixed-effects ordinal models?
Please provide justification for this choice and report sensitivity analyses using an ordinal mixed-effects model with random effects for both surgeon and scenario.

**Response/Changes: Additional explanation of statistical analysis has been added to the methods section of the manuscript.**

6. Could unmeasured covariates explain the observed regional effect?
Please clarify whether additional variables—such as country, subspecialty, prior training in EBM/Bayesian reasoning, or native language—were considered or included in extended models.

**Response: Yes, because the subgroups are small and non-representative. The SOVG mostly consists of white American or European men, which is why we believe that analyzing these variables would be unrepresentative. Due to this fact, we chose not to analyze these in multivariable analysis.**

**Changes: We now completely omit analysis of surgeon variables.**

7. PLOS ONE requires open data.
The statement “Data available on request” is not compliant and conflicts with the declaration “No – some restrictions will apply.”
Please clarify:
- What specific restrictions you intended
- How these restrictions align with PLOS ONE’s open-data policies
- How the dataset will be made publicly available

**Response/Changes: We provided the deidentified data with this revision.**

8. Additional demographic details appear to be missing.
For example, surgeons with 15 years of practice experience may differ significantly depending on age or career stage, and it is unclear whether responses differed by gender.
Please provide all relevant demographic variables and describe whether they influenced the results.

**Response/Changes: See Response to your comment 6. The analysis of surgeon variables was omitted.**

9. Please report the results of normality testing.
Since means and standard deviations (SD) should not be used for non-normally distributed data, include the outcomes of normality tests and consider presenting medians and IQRs if appropriate.

**Response/Changes: Results of normality testing were added.**

10. Scoring validity:
Please provide the explicit normative Bayesian calculations (priors, likelihoods, posteriors, and decision thresholds) used to justify the ranking of the response options.
If the ranking is based on qualitative Bayesian principles rather than exact numerical calculations, specify the criteria used to determine which options are “more Bayesian.”

**Response/Changes: A scoring rubric has been added as an appendix.**

Reviewer #2: Technical Soundness & Data Support

No actionable recommendations.

Reviewer #3: General comment

1. Are the musculoskeletal surgeons limited to only orthopaedic surgeons? Additionally, how are the distributions of subspecialties or fellowships among surgeons? Would this affect the answer to a specific scenario based on their subspecialty?

**Response/Changes: Clarified the description of the participants. Surgeons that address musculoskeletal pathophysiology include orthopedic surgeons, trauma (general) surgeons in Europe, and plastic surgeons.**

**2.** Some grammatical errors are found in the text. Please proofread and edit your manuscript before submission. Numbers below 11 should be written in text, not numerically.

**Response: Whether or not to write out numbers smaller than 10 is a style issue.**

**Changes: Manuscript has been proofread and edited. We will wait for the copy editing regarding writing out small numbers.**

**3.** Missing the tables of Scenario 6, 7, and 8. Furthermore, please number the scenarios in the table consecutively.

**Response/Changes: See response to reviewer 1, comment 2.**

4. Missing citation in line 342.

**Response/Changes: Added citation.**

5. Please complete the proper citation of citation no. 16.

**Response/Changes: Completed citation.**

6. 12/33 references were outdated; please use more valid references for the study.

**Response: The date of the reference is not relevant to its validity.**

**Changes: We looked at the older references and determined if there was a more instructive or useful newer reference we could add. We kept the key classic works that are highly relevant.**

7. How would the partial responders affect the validity of the study? A sensitivity analysis restricted to complete responders would help demonstrate that the main findings are robust.

**Response: In a scenario-based experiment, incomplete or unanswered scenarios do not alter the analysis any more than participation rate.**

**Changes: Additional explanation added to the methods and the limitations.**

8. Describe more of the scoring rubric used in this study. Please be transparent on how the author validated the ranking of responses from 1 to 4.

**Response/Changes: See response to Reviewer 1, comment 4.**

9. How did the author curate and validate the scenarios? Please explain more to make it reproducible.

**Response/Changes: See Response to Reviewer 1, Comment 4.**

10. Please clarify the writing of “95% CI: 2.82, 2.94” in lines 183-184.

**Response/Changes: This part was removed as surgeon characteristics were removed from analysis.**

11. The author should highlight the significance of the result of the association of the region of practice more (i.e., “significantly lower”), in lines 197-199.

**Response/Changes: Surgeon characteristics were removed from analysis**

12. Please include the original survey results, the scoring rubric, and any analysis code (Stata files) in the appendix. The data-availability statement should be added.

**Response/Changes: Data provided and data-availability statement updated.**

13. Lacking discussion in comparing the current study with previous or similar studies

**Response/Changes: The discussion may seem brief compared to discussion in other fields, but in our field, a discussion is complete when all the relevant points are adequately addressed. We confirmed that this was the case in our view. We are open to suggestions on content rather than length.**

14. Please elaborate further on the sample’s generalizability, as the sample in this study is drawn from a single group.

**Response/Changes: We addressed the comments on generalizability in the methods and limitations to make sure they were clear and complete.**

15. Consider adding a concise narrative or table summarising key prior studies (population, clinical domain, task type, main outcome, and typical proportion of Bayesian-consistent responses), and clearly state how the present findings align with or diverge from those patterns.

**Response: To our knowledge, there are currently no prior studies assessing Bayesian Reasoning among surgeons. We have addressed related studies about management of uncertainty and awareness of cognitive bias.**

**Changes: No changes**

16. For further implication, provide concrete examples of how Bayesian concepts could be integrated into orthopaedic training (e.g., structured case conferences emphasising priors and likelihood ratios, incorporation into fracture conference, use of decision aids for imaging and surgery thresholds.

**Response/Changes: Added additional suggestions for helping musculoskeletal surgeons become more facile at Bayesian reasoning.**

Reviewer #4:

1. Based on the research team's theoretical framework, the author assigned scores ranging from 1 to 4 to each response option. However, it remains unclear whether inter-rater reliability (IRR) was formally assessed. Furthermore, could the author clarify if this specific scoring rubric is substantiated by extant literature or established validation studies?

**Response/Changes: See Response to Reviewer 1, Comment 4.**

2. The authors report significant variations in reasoning patterns across different clinical scenarios. However, it remains unclear whether these discrepancies stem from genuine shifts in reasoning strategies or are artifacts of scenario-specific factors, such as task difficulty, situational familiarity, or relevance to specific subspecialties. Please clarify whether the equivalence of the scenarios was established during the study design. Furthermore, please specify if, and how, potential confounding effects, such as the 'difficulty effect' or 'familiarity bias,' were controlled for or mitigated.

**Response: We did not aim for psychometric equivalence across scenarios; rather, we prioritized ecological validity by designing vignettes that reflect the heterogeneity of actual clinical practice.**

**To mitigate potential confounding by 'difficulty' or 'subspecialty bias,' we employed the following strategies:**

**Iterative pilot testing: The scenarios were vetted by a diverse group of local surgeons to ensure the clinical content was accessible to general musculoskeletal surgeons, regardless of sub-specialization (e.g., avoiding esoteric pathology).**

**Focus on structure over content: The answer choices focused on the structure of the reasoning process (e.g., updating priors based on new data) rather than factual clinical knowledge. Thus, even if a surgeon was less familiar with a specific pathology, the logic required to answer the question remained accessible.**

**Finally, we interpret the observed variation across scenarios not as an artifact, but as a key finding: Bayesian reasoning in this cohort appears to be context-dependent rather than a static cognitive trait. Our use of mixed-effects modeling in the revised analysis further accounts for this variance by treating the scenario as a fixed effect.**

**Changes: Additional explanation has been added in the methods.**

3. The authors report statistically significant disparities in Bayesian reasoning scores across different regions; however, a concrete explanatory framework for these findings is currently absent. The authors are requested to clarify whether such variations might reflect regional differences in training systems, clinical guidelines, or the contextual relevance of the scenarios. Furthermore, please address whether the scenarios maintain equivalent representativeness across the various geographical regions included in the study.

**Response: We believe this demonstrates that differences can be detected, but these particular differences are likely confounded and should not be over-interpreted. Please also see our response at Reviewer 1 comment 6.**

**Changes: No changes**
