# Supplementary material for: Musculoskeletal surgeons use mixed reasoning rather than pure Bayesian strategies in clinical practice
Source: PLoS One. 2026 Jun 12;21(6):e0351694. doi: 10.1371/journal.pone.0351694 (PMC13262866; doi:10.1371/journal.pone.0351694)
Supplement: Attachment — Submitted filename: Response_to_Reviewers_auresp_2.docx [file pone.0351694.s003.docx]

Dear Editors of PLOS ONE,

Thank you for the careful review of our supporting dataset and for highlighting the concern regarding identifiable information in the originally submitted S1 file.

The originally submitted dataset contained no direct identifiers: no participant names, contact information, IP addresses, geolocation data, free-text responses, or institutional affiliations. The only field that could in principle be linked to an individual respondent was the platform-internal Qualtrics ResponseId, a randomly generated alphanumeric string used by the survey platform to index responses internally. While the inclusion of these identifiers is common practice in published survey datasets, we recognize and appreciate PLOS ONE’s higher standard for de-identification.

We have therefore replaced the Qualtrics ResponseId column with sequential integers (1–153). All other variables — the demographic items (Q2–Q6), the eight scenario response items, and the composite SC0 score — remain unchanged from the originally submitted file. The structure, sheet name, column names, and codings are identical, ensuring full reproducibility of all analyses reported in the manuscript using the accompanying Python analysis script.

The revised S1 Dataset is attached. We are happy to provide any further clarification the editorial office may require.

Sincerely,

David Ring
