# Supplementary material for: Musculoskeletal surgeons use mixed reasoning rather than pure Bayesian strategies in clinical practice
Source: PLoS One. 2026 Jun 12;21(6):e0351694. doi: 10.1371/journal.pone.0351694 (PMC13262866; doi:10.1371/journal.pone.0351694)
Supplement: Peer review history

# Peer review: Decision Letter 0

**DOI:** 10.1371/journal.pone.0351694.r001

**Authors:** Ismail Badr

30 Dec 2025

PONE-D-25-55463Musculoskeletal Surgeons Use Mixed Reasoning Rather Than Pure Bayesian Strategies in

Clinical PracticePLOS One

Dear Dr. Ring,

Thank you for submitting your manuscript to PLOS ONE. After careful consideration, we feel that it has merit but does not fully meet PLOS ONE’s publication criteria as it currently stands. Therefore, we invite you to submit a revised version of the manuscript that addresses the points raised during the review process.

Please submit your revised manuscript by Feb 12 2026 11:59PM. If you will need more time than this to complete your revisions, please reply to this message or contact the journal office at plosone@plos.org. When you're ready to submit your revision, log on to https://www.editorialmanager.com/pone/ and select the 'Submissions Needing Revision' folder to locate your manuscript file.

Please include the following items when submitting your revised manuscript:

- A letter that responds to each point raised by the academic editor and reviewer(s). You should upload this letter as a separate file labeled 'Response to Reviewers'.
- A marked-up copy of your manuscript that highlights changes made to the original version. You should upload this as a separate file labeled 'Revised Manuscript with Track Changes'.
- An unmarked version of your revised paper without tracked changes. You should upload this as a separate file labeled 'Manuscript'.

If you would like to make changes to your financial disclosure, please include your updated statement in your cover letter. Guidelines for resubmitting your figure files are available below the reviewer comments at the end of this letter.

If applicable, we recommend that you deposit your laboratory protocols in protocols.io to enhance the reproducibility of your results. Protocols.io assigns your protocol its own identifier (DOI) so that it can be cited independently in the future. For instructions see: https://journals.plos.org/plosone/s/submission-guidelines#loc-laboratory-protocols. Additionally, PLOS ONE offers an option for publishing peer-reviewed Lab Protocol articles, which describe protocols hosted on protocols.io. Read more information on sharing protocols at https://plos.org/protocols?utm_medium=editorial-email&utm_source=authorletters&utm_campaign=protocols.

We look forward to receiving your revised manuscript.

Kind regards,

Ismail Tawfeek Abdelaziz Badr, M.D.

Academic Editor

PLOS One

Journal Requirements:

When submitting your revision, we need you to address these additional requirements.

1. Please ensure that your manuscript meets PLOS ONE's style requirements, including those for file naming. The PLOS ONE style templates can be found at

https://journals.plos.org/plosone/s/file?id=wjVg/PLOSOne_formatting_sample_main_body.pdf and

https://journals.plos.org/plosone/s/file?id=ba62/PLOSOne_formatting_sample_title_authors_affiliations.pdf.

2. Please provide additional details regarding participant consent. In the ethics statement in the Methods and online submission information, please ensure that you have specified what type you obtained (for instance, written or verbal, and if verbal, how it was documented and witnessed). If your study included minors, state whether you obtained consent from parents or guardians. If the need for consent was waived by the ethics committee, please include this information.

3. We note that you have indicated that there are restrictions to data sharing for this study. For studies involving human research participant data or other sensitive data, we encourage authors to share de-identified or anonymized data. However, when data cannot be publicly shared for ethical reasons, we allow authors to make their data sets available upon request. For information on unacceptable data access restrictions, please see http://journals.plos.org/plosone/s/data-availability#loc-unacceptable-data-access-restrictions.

Before we proceed with your manuscript, please address the following prompts:

a) If there are ethical or legal restrictions on sharing a de-identified data set, please explain them in detail (e.g., data contain potentially identifying or sensitive patient information, data are owned by a third-party organization, etc.) and who has imposed them (e.g., a Research Ethics Committee or Institutional Review Board, etc.). Please also provide contact information for a data access committee, ethics committee, or other institutional body to which data requests may be sent.

b) If there are no restrictions, please upload the minimal anonymized data set necessary to replicate your study findings to a stable, public repository and provide us with the relevant URLs, DOIs, or accession numbers. Please see http://www.bmj.com/content/340/bmj.c181.long for guidelines on how to de-identify and prepare clinical data for publication. For a list of recommended repositories, please see https://journals.plos.org/plosone/s/recommended-repositories. You also have the option of uploading the data as Supporting Information files, but we would recommend depositing data directly to a data repository if possible.

Please update your Data Availability statement in the submission form accordingly.

4. In the online submission form you indicate that your data is not available for proprietary reasons and have provided a contact point for accessing this data. Please note that your current contact point is a co-author on this manuscript. According to our Data Policy, the contact point must not be an author on the manuscript and must be an institutional contact, ideally not an individual. Please revise your data statement to a non-author institutional point of contact, such as a data access or ethics committee, and send this to us via return email. Please also include contact information for the third party organization, and please include the full citation of where the data can be found.

5. If the reviewer comments include a recommendation to cite specific previously published works, please review and evaluate these publications to determine whether they are relevant and should be cited. There is no requirement to cite these works unless the editor has indicated otherwise.

[Note: HTML markup is below. Please do not edit.]

Reviewers' comments:

Reviewer's Responses to Questions

**Comments to the Author**

1. Is the manuscript technically sound, and do the data support the conclusions?

The manuscript must describe a technically sound piece of scientific research with data that supports the conclusions. Experiments must have been conducted rigorously, with appropriate controls, replication, and sample sizes. The conclusions must be drawn appropriately based on the data presented.

Reviewer #1: Partly

Reviewer #2: Yes

Reviewer #3: Yes

Reviewer #4: Yes

**********

2. Has the statistical analysis been performed appropriately and rigorously?

Reviewer #1: No

Reviewer #2: Yes

Reviewer #3: Yes

Reviewer #4: Yes

**********

3. Have the authors made all data underlying the findings in their manuscript fully available?

The PLOS Data policy requires authors to make all data underlying the findings described in their manuscript fully available without restriction, with rare exception (please refer to the Data Availability Statement in the manuscript PDF file). The data should be provided as part of the manuscript or its supporting information, or deposited to a public repository. For example, in addition to summary statistics, the data points behind means, medians and variance measures should be available. If there are restrictions on publicly sharing data—e.g. participant privacy or use of data from a third party—those must be specified.

Reviewer #1: No

Reviewer #2: Yes

Reviewer #3: No

Reviewer #4: Yes

**********

4. Is the manuscript presented in an intelligible fashion and written in standard English?

PLOS ONE does not copyedit accepted manuscripts, so the language in submitted articles must be clear, correct, and unambiguous. Any typographical or grammatical errors should be corrected at revision, so please note any specific errors here.

Reviewer #1: Yes

Reviewer #2: Yes

Reviewer #3: Yes

Reviewer #4: Yes

**********

5. Review Comments to the Author

Please use the space provided to explain your answers to the questions above. You may also include additional comments for the author, including concerns about dual publication, research ethics, or publication ethics. (Please upload your review as an attachment if it exceeds 20,000 characters)

Reviewer #1: • Did you perform any test to determine the appropriate sample size?

Given the sensitivity of the study, please revise the manuscript to include complete details on sample size justification.

• Please provide the full text of all eight scenarios, including the four response options for each scenario, as well as the detailed normative Bayesian justification (priors, likelihoods, posteriors, and the criteria used to rank options from non-Bayesian to fully Bayesian).

Only Scenarios 1, 2, 4, and 5 appear to be included; the full set of eight scenarios is not visible.

• In the section on “other relevant findings,” the references to Scenario 1 (Table 3) and Scenario 2 (Table 4) appear inaccurate.

Please review and correct the scenario numbering and corresponding table references.

• How were the scenarios and their Bayesian rankings developed and validated?

Please indicate whether you conducted:

- Expert panel review

- Pilot testing

- Cognitive interviews

- Interrater agreement assessments for the Bayesian ranking criteria

• Given that the primary outcome is ordinal, why did you choose linear models instead of ordinal or mixed-effects ordinal models?

Please provide justification for this choice and report sensitivity analyses using an ordinal mixed-effects model with random effects for both surgeon and scenario.

• Could unmeasured covariates explain the observed regional effect?

Please clarify whether additional variables—such as country, subspecialty, prior training in EBM/Bayesian reasoning, or native language—were considered or included in extended models.

• PLOS ONE requires open data.

The statement “Data available on request” is not compliant and conflicts with the declaration “No – some restrictions will apply.”

Please clarify:

- What specific restrictions you intended

- How these restrictions align with PLOS ONE’s open-data policies

- How the dataset will be made publicly available

•Additional demographic details appear to be missing.

For example, surgeons with 15 years of practice experience may differ significantly depending on age or career stage, and it is unclear whether responses differed by gender.

Please provide all relevant demographic variables and describe whether they influenced the results.

• Please report the results of normality testing.

Since means and standard deviations (SD) should not be used for non-normally distributed data, include the outcomes of normality tests and consider presenting medians and IQRs if appropriate.

• Scoring validity:

Please provide the explicit normative Bayesian calculations (priors, likelihoods, posteriors, and decision thresholds) used to justify the ranking of the response options.

If the ranking is based on qualitative Bayesian principles rather than exact numerical calculations, specify the criteria used to determine which options are “more Bayesian.”

Reviewer #2: Technical Soundness & Data Support

The manuscript is technically sound, and the presented data robustly support the conclusions. The experimental design and methodology are appropriate for the research objectives.

Statistical Analysis

The statistical analysis has been performed appropriately and rigorously. The methods are well-described, and the results are presented with sufficient detail to validate the findings.

Data Availability

The authors have made all underlying data fully available, ensuring transparency and reproducibility of the study.

Clarity & Language

The manuscript is well-structured, clearly written in standard English, and presented in an intelligible manner. The logical flow enhances readability.

Overall Recommendation:

The manuscript meets the expected scientific and editorial standards for publication.

Reviewer #3: General comment

1. This study is important and novel in the field of surgical decision-making. However, some methodological transparency issues must be addressed to meet the journal’s standard.

2. Are the musculoskeletal surgeons limited to only orthopaedic surgeons? Additionally, how are the distributions of subspecialties or fellowships among surgeons? Would this affect the answer to a specific scenario based on their subspecialty?

3. Some grammatical errors are found in the text. Please proofread and edit your manuscript before submission. Numbers below 11 should be written in text, not numerically.

4. Missing the tables of Scenario 6, 7, and 8. Furthermore, please number the scenarios in the table consecutively.

5. Missing citation in line 342.

6. Please complete the proper citation of citation no. 16.

7. 12/33 references were outdated; please use more valid references for the study.

Methods

1. How would the partial responders affect the validity of the study? A sensitivity analysis restricted to complete responders would help demonstrate that the main findings are robust.

2. Describe more of the scoring rubric used in this study. Please be transparent on how the author validated the ranking of responses from 1 to 4.

3. How did the author curate and validate the scenarios? Please explain more to make it reproducible.

Results

1. Please clarify the writing of “95% CI: 2.82, 2.94” in lines 183-184.

2. The author should highlight the significance of the result of the association of the region of practice more (i.e., “significantly lower”), in lines 197-199.

3. Please include the original survey results, the scoring rubric, and any analysis code (Stata files) in the appendix. The data-availability statement should be added.

Discussion

1. Lacking discussion in comparing the current study with previous or similar studies

2. Please elaborate further on the sample’s generalizability, as the sample in this study is drawn from a single group.

3. Consider adding a concise narrative or table summarising key prior studies (population, clinical domain, task type, main outcome, and typical proportion of Bayesian-consistent responses), and clearly state how the present findings align with or diverge from those patterns.

4. For further implication, provide concrete examples of how Bayesian concepts could be integrated into orthopaedic training (e.g., structured case conferences emphasising priors and likelihood ratios, incorporation into fracture conference, use of decision aids for imaging and surgery thresholds.

Overall, the manuscript requires major revision to be considered for publication in this journal.

Reviewer #4: This manuscript addresses an important topic in clinical decision-making and presents a carefully designed scenario-based assessment of Bayesian reasoning among musculoskeletal surgeons. The study is generally well conducted and clearly reported, and the findings provide useful insight into the context-dependent nature of clinical reasoning under uncertainty. However, several aspects of the study designand interpretation would benefit from further clarification, as outlined below.

1.Based on the research team's theoretical framework, the author assigned scores ranging from 1 to 4 to each response option. However, it remains unclear whether inter-rater reliability (IRR) was formally assessed. Furthermore, could the author clarify if this specific scoring rubric is substantiated by extant literature or established validation studies?

2.The authors report significant variations in reasoning patterns across different clinical scenarios. However, it remains unclear whether these discrepancies stem from genuine shifts in reasoning strategies or are artifacts of scenario-specific factors, such as task difficulty, situational familiarity, or relevance to specific subspecialties. Please clarify whether the equivalence of the scenarios was established during the study design. Furthermore, please specify if, and how, potential confounding effects, such as the 'difficulty effect' or 'familiarity bias,' were controlled for or mitigated.

3.The authors report statistically significant disparities in Bayesian reasoning scores across different regions; however, a concrete explanatory framework for these findings is currently absent. The authors are requested to clarify whether such variations might reflect regional differences in training systems, clinical guidelines, or the contextual relevance of the scenarios. Furthermore, please address whether the scenarios maintain equivalent representativeness across the various geographical regions included in the study.

**********

6. PLOS authors have the option to publish the peer review history of their article (what does this mean?). If published, this will include your full peer review and any attached files.

If you choose “no”, your identity will remain anonymous but your review may still be made public.

**Do you want your identity to be public for this peer review?** For information about this choice, including consent withdrawal, please see our Privacy Policy.

Reviewer #1: **Yes:** Sameh Eldaly

Reviewer #2: No

Reviewer #3: **Yes:** Ismail Hadisoebroto Dilogo

Reviewer #4: No

**********

[NOTE: If reviewer comments were submitted as an attachment file, they will be attached to this email and accessible via the submission site. Please log into your account, locate the manuscript record, and check for the action link "View Attachments". If this link does not appear, there are no attachment files.]

To ensure your figures meet our technical requirements, please review our figure guidelines: https://journals.plos.org/plosone/s/figures

You may also use PLOS’s free figure tool, NAAS, to help you prepare publication quality figures: https://journals.plos.org/plosone/s/figures#loc-tools-for-figure-preparation.

NAAS will assess whether your figures meet our technical requirements by comparing each figure against our figure specifications.

# Author comment: Author response to Decision Letter 1

**DOI:** 10.1371/journal.pone.0351694.r002

29 Jan 2026

Please see the "response to reviewers" file added to this revision.

Attachment Submitted filename: Response to Reviewers.docx

# Peer review: Decision Letter 1

**DOI:** 10.1371/journal.pone.0351694.r003

**Authors:** Ismail Badr

24 Mar 2026

PONE-D-25-55463R1Musculoskeletal Surgeons Use Mixed Reasoning Rather Than Pure Bayesian Strategies in Clinical PracticePLOS One

Dear Dr. Ring,

Thank you for submitting your manuscript to PLOS ONE. After careful consideration, we feel that it has merit but does not fully meet PLOS ONE’s publication criteria as it currently stands. Therefore, we invite you to submit a revised version of the manuscript that addresses the points raised during the review process.

Please submit your revised manuscript by May 07 2026 11:59PM. If you will need more time than this to complete your revisions, please reply to this message or contact the journal office at plosone@plos.org. When you're ready to submit your revision, log on to https://www.editorialmanager.com/pone/ and select the 'Submissions Needing Revision' folder to locate your manuscript file.

Please include the following items when submitting your revised manuscript:

- A letter that responds to each point raised by the academic editor and reviewer(s). You should upload this letter as a separate file labeled 'Response to Reviewers'.
- A marked-up copy of your manuscript that highlights changes made to the original version. You should upload this as a separate file labeled 'Revised Manuscript with Track Changes'.
- An unmarked version of your revised paper without tracked changes. You should upload this as a separate file labeled 'Manuscript'.

If you would like to make changes to your financial disclosure, please include your updated statement in your cover letter. Guidelines for resubmitting your figure files are available below the reviewer comments at the end of this letter.

If applicable, we recommend that you deposit your laboratory protocols in protocols.io to enhance the reproducibility of your results. Protocols.io assigns your protocol its own identifier (DOI) so that it can be cited independently in the future. For instructions see: https://journals.plos.org/plosone/s/submission-guidelines#loc-laboratory-protocols. Additionally, PLOS ONE offers an option for publishing peer-reviewed Lab Protocol articles, which describe protocols hosted on protocols.io. Read more information on sharing protocols at https://plos.org/protocols?utm_medium=editorial-email&utm_source=authorletters&utm_campaign=protocols.

We look forward to receiving your revised manuscript.

Kind regards,

Ismail Tawfeek Abdelaziz Badr

Academic Editor

PLOS One

Journal Requirements:

If the reviewer comments include a recommendation to cite specific previously published works, please review and evaluate these publications to determine whether they are relevant and should be cited. There is no requirement to cite these works unless the editor has indicated otherwise.

[Note: HTML markup is below. Please do not edit.]

Reviewers' comments:

Reviewer's Responses to Questions

**Comments to the Author**

1. If the authors have adequately addressed your comments raised in a previous round of review and you feel that this manuscript is now acceptable for publication, you may indicate that here to bypass the “Comments to the Author” section, enter your conflict of interest statement in the “Confidential to Editor” section, and submit your "Accept" recommendation.

Reviewer #1: (No Response)

Reviewer #3: All comments have been addressed

Reviewer #4: All comments have been addressed

Reviewer #5: All comments have been addressed

Reviewer #6: (No Response)

Reviewer #7: (No Response)

**********

2. Is the manuscript technically sound, and do the data support the conclusions?

The manuscript must describe a technically sound piece of scientific research with data that supports the conclusions. Experiments must have been conducted rigorously, with appropriate controls, replication, and sample sizes. The conclusions must be drawn appropriately based on the data presented.

Reviewer #1: (No Response)

Reviewer #3: Yes

Reviewer #4: Yes

Reviewer #5: Yes

Reviewer #6: (No Response)

Reviewer #7: Yes

**********

3. Has the statistical analysis been performed appropriately and rigorously?

Reviewer #1: No

Reviewer #3: N/A

Reviewer #4: Yes

Reviewer #5: N/A

Reviewer #6: (No Response)

Reviewer #7: Yes

**********

4. Have the authors made all data underlying the findings in their manuscript fully available?

The PLOS Data policy requires authors to make all data underlying the findings described in their manuscript fully available without restriction, with rare exception (please refer to the Data Availability Statement in the manuscript PDF file). The data should be provided as part of the manuscript or its supporting information, or deposited to a public repository. For example, in addition to summary statistics, the data points behind means, medians and variance measures should be available. If there are restrictions on publicly sharing data—e.g. participant privacy or use of data from a third party—those must be specified.

Reviewer #1: Yes

Reviewer #3: Yes

Reviewer #4: Yes

Reviewer #5: Yes

Reviewer #6: (No Response)

Reviewer #7: Yes

**********

5. Is the manuscript presented in an intelligible fashion and written in standard English?

PLOS ONE does not copyedit accepted manuscripts, so the language in submitted articles must be clear, correct, and unambiguous. Any typographical or grammatical errors should be corrected at revision, so please note any specific errors here.

Reviewer #1: No

Reviewer #3: Yes

Reviewer #4: Yes

Reviewer #5: Yes

Reviewer #6: (No Response)

Reviewer #7: Yes

**********

6. Review Comments to the Author

Please use the space provided to explain your answers to the questions above. You may also include additional comments for the author, including concerns about dual publication, research ethics, or publication ethics. (Please upload your review as an attachment if it exceeds 20,000 characters)

Reviewer #1: (No Response)

Reviewer #3: • Please provide more information about the participant characteristics in the abstract, such as the regions and years of experience.

• Please clarify how the responses were obtained from the study subjects. Was it through an online questionnaire or a multicenter study?

• How were younger musculoskeletal surgeons represented in this study? Most of the participants seem to have several years of experience in their field, which creates an imbalance between junior and senior surgeons. This may introduce a bias in the study.

• Please add an explanation regarding the partial responses. Could these affect the results and introduce bias? Or address this in the study limitations.

• Please include citations for lines 225 to 248.

• Please explain how your findings compare with previous studies in the literature.

• Were there any regional differences in the findings among orthopaedic surgeons? Please expand the discussion on these regional variations.

Reviewer #4: (No Response)

Reviewer #5: I found the topic is interesting, original but difficult to interpret and analyze. This study addresses an important and underexplored aspect of surgical decision-making.

The Cronbach alpha of 0.43 suggests low internal consistency. While the authors interpret this as evidence of context-dependent reasoning, it also raises concern that the instrument may not be measuring a unified construct. The manuscript would benefit from deeper discussion of whether “Bayesian reasoning” is being operationalized consistently across scenarios and whether the low alpha reflects measurement limitations rather than cognitive variability.

The central conclusion that surgeons use mixed reasoning rather than pure Bayesian strategies may be overstated. Mixed reasoning could reflect adaptive expertise rather than deviation from a normative ideal. The discussion should more clearly distinguish between cognitive flexibility and suboptimal probabilistic reasoning.

The convenience sample from the Science of Variation Group (predominantly academic, highly experienced surgeons) limits external validity. This limitation should be emphasized more clearly in the Discussion and Conclusions.

Reviewer #6: This study investigates the cognitive logic of 153 senior musculoskeletal surgeons (primarily from North America and Europe) when navigating clinical uncertainty. Through eight simulated clinical scenarios, the authors assessed the extent to which surgeons employ Bayesian reasoning in diagnostic and treatment decisions. The findings suggest that surgical decision-making is not purely probability-driven but rather characterized by "mixed reasoning," which is highly context-dependent. The revised manuscript appropriately introduced mixed-effects modeling to further analyze the heterogeneity across scenarios.

1. Abstract

The authors are encouraged to provide a clearer operational definition of "Mixed Reasoning" within the context of this study. The conclusion should explicitly state the implications of this logical instability for the implementation of clinical guidelines.

2. Introduction

Strengthen the link between "logical flaws" and "adverse clinical outcomes." Adding literature regarding common cognitive biases in orthopaedics (e.g., overdiagnosis or diagnostic anchoring) would better justify the necessity of studying reasoning logic as a tool to prevent over-medicalization or unnecessary surgery.

3. Methods

Please clarify whether "individual surgeon" was treated as a random effect in the mixed-effects model. While scenarios are treated as fixed effects, it is crucial to determine if the model adequately separates variance caused by the scenarios from variance stemming from individual cognitive differences or professional backgrounds.

4. Results

Although the authors view regional differences as potential confounders, providing a subgroup analysis by region (e.g., North America vs. Europe) in the supplementary materials is recommended. Differences in medical education systems regarding statistical literacy may exist; showcasing these trends—even without over-interpretation—offers valuable insights for a global audience.

5. Discussion

Expand the discussion on how "non-Bayesian logic" practically impacts the quality of care. For instance, does failing to follow Bayesian logic in low-prevalence scenarios significantly increase false-positive rates? The manuscript needs to bridge the gap between "abstract logic scores" and "tangible clinical risks."

Provide more actionable recommendations for musculoskeletal education. Given the "context-dependent" nature of reasoning, education should move beyond teaching formulas toward Case-Based Learning (CBL) to correct cognitive biases within specific clinical contexts.

6. Conclusion

Emphasize the finding that "extensive clinical experience does not necessarily equate to logical rigor." This serves as a critical policy reminder for Continuing Medical Education (CME) programs targeting senior clinicians.

7. References

The timeliness of the references is acceptable, but it is recommended to supplement the basic literature on the application of mixed-effects models in cognitive research, as well as the latest studies on surgeon decision preferences (Surgeon preference) in the past two years (2024-2025), in order to demonstrate that this research is at the forefront of the field.

Reviewer #7: Short Formal Peer Review (150–200 words)

This manuscript investigates how musculoskeletal surgeons apply Bayesian reasoning when making clinical decisions under uncertainty. Using a vignette-based survey, a group of 153 musculoskeletal surgeons responded to eight clinical scenarios designed to reflect varying levels of Bayesian reasoning. The results suggest that surgeons use a mixture of Bayesian and non-Bayesian reasoning strategies, with substantial variability across scenarios. The study highlights that surgeons appear capable of Bayesian reasoning but apply it selectively depending on the clinical context.

The topic is relevant and timely, particularly given the increasing emphasis on probabilistic reasoning in medical education and the emergence of AI-supported clinical decision tools. The vignette-based approach provides a practical method for exploring reasoning patterns, and the study offers useful insights into how clinicians interpret evidence and update diagnostic probabilities.

Overall, the manuscript addresses an important aspect of clinical reasoning and contributes valuable observations. I see no issues with this manuscript.

**********

7. PLOS authors have the option to publish the peer review history of their article (what does this mean?). If published, this will include your full peer review and any attached files.

If you choose “no”, your identity will remain anonymous but your review may still be made public.

**Do you want your identity to be public for this peer review?** For information about this choice, including consent withdrawal, please see our Privacy Policy.

Reviewer #1: **Yes:** Sameh Eldaly

Reviewer #3: No

Reviewer #4: No

Reviewer #5: No

Reviewer #6: No

Reviewer #7: No

**********

[NOTE: If reviewer comments were submitted as an attachment file, they will be attached to this email and accessible via the submission site. Please log into your account, locate the manuscript record, and check for the action link "View Attachments". If this link does not appear, there are no attachment files.]

To ensure your figures meet our technical requirements, please review our figure guidelines: https://journals.plos.org/plosone/s/figures

You may also use PLOS’s free figure tool, NAAS, to help you prepare publication quality figures: https://journals.plos.org/plosone/s/figures#loc-tools-for-figure-preparation.

NAAS will assess whether your figures meet our technical requirements by comparing each figure against our figure specifications.

# Author comment: Author response to Decision Letter 2

**DOI:** 10.1371/journal.pone.0351694.r004

30 Apr 2026

Response to Reviewers

Manuscript: Musculoskeletal Surgeons Use Mixed Reasoning Rather Than Pure Bayesian Strategies in Clinical Practice

Journal: PLOS ONE

We thank the editors and reviewers for their thoughtful and constructive comments on our manuscript. We have carefully addressed each point below. Reviewer comments are shown in italics; our responses follow. All line numbers refer to the revised clean manuscript.

Reviewer #3

Please provide more information about the participant characteristics in the abstract, such as the regions and years of experience.

Response: We have updated the abstract Methods sentence to include specific percentages: 58% North America, 30% Europe, and 69% with over 15 years in practice.

Manuscript change: Abstract Methods sentence updated to include regional and experience percentages.

Please clarify how the responses were obtained from the study subjects. Was it through an online questionnaire or a multicenter study?

Response: We have updated the abstract Methods sentence and the Study design and setting paragraph to specify that data were collected via an online survey distributed to members of the Science of Variation Group (SOVG) in December 2024.

Manuscript change: Abstract Methods sentence and Study design and setting paragraph updated to clarify online survey administration.

How were younger musculoskeletal surgeons represented in this study? Most of the participants seem to have several years of experience in their field, which creates an imbalance between junior and senior surgeons. This may introduce a bias in the study.

Response: This is a valid observation. The SOVG membership is predominantly experienced, academic surgeons, which necessarily limits representation of early-career clinicians. We have added a sentence to the Limitations section noting that with only 3.5% of participants having fewer than 5 years in practice, results may not reflect the full spectrum of clinical reasoning across career stages.

Manuscript change: One sentence added to Limitations paragraph addressing junior surgeon underrepresentation and its directional effect on results.

Please add an explanation regarding the partial responses. Could these affect the results and introduce bias? Or address this in the study limitations.

Response: We have added a sentence to the Limitations section noting that including the 18 partial responses using per-scenario averages assumes responses are missing at random. If non-completion is related to reasoning difficulty or discomfort with certain scenarios, this could introduce bias, though the direction of such bias is unclear.

Manuscript change: One sentence added to Limitations addressing partial responses and the missing-at-random assumption.

Please include citations for lines 225 to 248.

Response: We have added appropriate citations to the Discussion background paragraphs, drawing on references already present in the manuscript: Teunis et al. 2016 [23]; Janssen et al. 2021 [21]; Croskerry 2009 [24]; Norman & Eva 2010 [28].

Manuscript change: Citations added to Discussion background section.

Please explain how your findings compare with previous studies in the literature.

Response: We have added a comparative paragraph to the Discussion. Specifically, we note that our finding of context-dependent mixed reasoning is consistent with Rottman et al. (2016, 2017) in residents, and that our base rate neglect findings parallel Manrai et al. (2014) and Teunis et al. (2016) — notably, only 11% of surgeons correctly applied base rate reasoning in the scaphoid scenario, strikingly similar to Teunis et al.’s finding. We also note that our median score of 3.0 is higher than purely non-Bayesian frameworks would predict, consistent with Croskerry’s adaptive expertise model.

Manuscript change: Comparative paragraph added to Discussion (Background, rationale, and general results section) with citations [9,10,17,23,29].

Were there any regional differences in the findings among orthopaedic surgeons? Please expand the discussion on these regional variations.

Response: Regional differences were identified: linear regression found slightly lower Bayesian reasoning scores among European participants (β = −0.23, 95% CI −0.41 to −0.055, p=0.011). We intentionally did not emphasize this finding in the abstract given the modest effect size, the small regional subgroup sizes, and the non-representative nature of the SOVG sample. We have added a sentence to the Discussion acknowledging the finding and contextualizing its limitations. We did not add a formal supplementary regional table, as the subgroup sizes outside North America and Europe are too small for meaningful comparison (South America n=10, Australia n=4, Asia n=2, Africa n=1), and we are concerned that such a table would give undue prominence to a secondary exploratory finding in a sample not designed to support regional comparisons.

Manuscript change: One sentence added to Discussion (end of “Do musculoskeletal surgeons employ Bayesian reasoning?” section) acknowledging the regional finding with appropriate caveats. Finding not added to abstract. No supplementary regional table added given small subgroup sizes.

Reviewer #5

The Cronbach alpha of 0.43 suggests low internal consistency. While the authors interpret this as evidence of context-dependent reasoning, it also raises concern that the instrument may not be measuring a unified construct. The manuscript would benefit from deeper discussion of whether “Bayesian reasoning” is being operationalized consistently across scenarios and whether the low alpha reflects measurement limitations rather than cognitive variability.

Response: We agree this is an important interpretive question. A Cronbach alpha of 0.43 is consistent with two explanations: first, that the scenarios inadequately operationalize a single unified construct; second, that Bayesian reasoning is genuinely context-dependent and not a stable individual trait. We cannot fully distinguish between these possibilities from survey data alone. We have revised the Discussion to acknowledge both interpretations explicitly, noting that the low alpha may reflect measurement heterogeneity across structurally different clinical scenarios, and that this ambiguity is itself a meaningful finding — it cautions against treating Bayesian reasoning as a unitary cognitive disposition amenable to simple measurement.

Manuscript change: Paragraph added to the “Is there variation” section of the Discussion acknowledging both interpretations of the low Cronbach alpha: measurement heterogeneity across structurally diverse scenarios, and genuine context-dependence of Bayesian reasoning as a construct. The ambiguity between these explanations is noted explicitly.

The central conclusion that surgeons use mixed reasoning rather than pure Bayesian strategies may be overstated. Mixed reasoning could reflect adaptive expertise rather than deviation from a normative ideal. The discussion should more clearly distinguish between cognitive flexibility and suboptimal probabilistic reasoning.

Response: We thank the reviewer for this important distinction. We agree that our original framing risked conflating mixed reasoning with suboptimal reasoning, which is not warranted. As Croskerry (2018) argues, adaptive expertise in clinical decision-making is characterized precisely by the ability to modulate reasoning strategy according to context — applying deliberate analytical reasoning when it is most needed and relying on pattern recognition when appropriate. Some of the non-Bayesian responses in our data almost certainly reflect genuine base rate neglect (the scaphoid scenario is a clear example, where only 11% of surgeons correctly applied the low prior probability). However, other instances of mixed or non-Bayesian responses may reflect legitimate contextual judgment rather than cognitive failure. Our data do not allow us to fully distinguish between these two phenomena. We have revised the Discussion and Conclusions to make this distinction explicit, acknowledging that mixed reasoning may reflect adaptive expertise in some contexts while representing a correctable bias in others.

Manuscript change: Paragraph added to the “Do musculoskeletal surgeons employ Bayesian reasoning?” section of the Discussion distinguishing cognitive flexibility from suboptimal probabilistic reasoning. Croskerry (2018) [29] cited explicitly. Language revised in Conclusions to avoid framing mixed reasoning as straightforwardly deficient.

The convenience sample from the Science of Variation Group (predominantly academic, highly experienced surgeons) limits external validity. This limitation should be emphasized more clearly in the Discussion and Conclusions.

Response: We agree. The SOVG sample — predominantly academic, highly experienced, and self-selected for engagement with research — represents a ceiling on external validity that we had not sufficiently emphasized. Paradoxically, this sample likely overestimates Bayesian reasoning relative to the average practicing surgeon, meaning the true prevalence of non-Bayesian reasoning in broader surgical populations may be higher than our data suggest. We have strengthened the limitation language in both the Limitations section and the Conclusions.

Manuscript change: Limitations section expanded with a dedicated paragraph on the non-representativeness of the SOVG sample and its directional effect on results. Conclusions revised to include a caveat on generalizability.

Reviewer #6

Abstract: The authors are encouraged to provide a clearer operational definition of “Mixed Reasoning” within the context of this study. The conclusion should explicitly state the implications of this logical instability for the implementation of clinical guidelines.

Response: We have added a brief operational definition of “Mixed Reasoning” to the abstract, consistent with the scoring rubric in Table 1: reasoning in which prior probability is acknowledged but underweighted, without explicit probabilistic updating. We have also added a sentence to the Conclusions noting that context-dependent reasoning variability has implications for clinical guideline implementation — specifically, that guidelines designed around normative probabilistic reasoning may be applied inconsistently when the underlying reasoning substrate varies by clinical context and individual surgeon.

Manuscript change: One parenthetical clause added to the abstract defining Mixed Reasoning operationally. One sentence added to Conclusions on clinical guideline implementation implications.

Introduction: Strengthen the link between “logical flaws” and “adverse clinical outcomes.” Adding literature regarding common cognitive biases in orthopaedics (e.g., overdiagnosis or diagnostic anchoring) would better justify the necessity of studying reasoning logic as a tool to prevent over-medicalization or unnecessary surgery.

Response: We agree that the Introduction would benefit from an explicit link between cognitive reasoning failures and adverse clinical outcomes. Janssen et al. (2021) on cognitive biases in orthopaedic surgery is already cited in this context. We have added one sentence to the Rationale section of the Introduction connecting non-Bayesian reasoning patterns — specifically base rate neglect and over-reliance on test results — to documented clinical consequences including unnecessary surgery, overdiagnosis, and failure to appropriately reassure patients in low-probability scenarios.

Manuscript change: One sentence added to the Rationale section of the Introduction linking non-Bayesian reasoning patterns explicitly to adverse clinical outcomes.

Methods: Please clarify whether “individual surgeon” was treated as a random effect in the mixed-effects model. While scenarios are treated as fixed effects, it is crucial to determine if the model adequately separates variance caused by the scenarios from variance stemming from individual cognitive differences or professional backgrounds.

Response: We appreciate this comment. To clarify: no mixed-effects model was included in the submitted manuscript. The reviewer may have encountered this reference in the context of the tracked-changes version submitted for review, which contained visible editorial notes and in-progress text that may have been misread as finalized methods. Our statistical approach used Shapiro-Wilk for normality testing, non-parametric descriptive statistics (median, IQR), Mann-Whitney U for regional comparisons, and linear regression for surgeon-level predictors. We have clarified the statistical methods paragraph to ensure no ambiguity.

Manuscript change: Statistical analysis paragraph reviewed and clarified for precision. No mixed-effects model was added.

Results: Although the authors view regional differences as potential confounders, providing a subgroup analysis by region (e.g., North America vs. Europe) in the supplementary materials is recommended.

Response: We have chosen not to add a formal subgroup analysis by region, as regional differences were not a primary aim of this study and the subgroup sizes outside North America and Europe are too small for meaningful comparison (South America n=10, Australia n=4, Asia n=2, Africa n=1). The linear regression finding for European participants (β = −0.23, p=0.011) is reported in the Results and discussed with appropriate caveats. We are concerned that a supplementary regional table would risk giving undue prominence to a secondary exploratory finding in a convenience sample not designed to support regional comparisons.

Manuscript change: No supplementary regional table added. Regional finding retained in Results with contextualizing language in Discussion.

Discussion: Expand the discussion on how “non-Bayesian logic” practically impacts the quality of care. Does failing to follow Bayesian logic in low-prevalence scenarios significantly increase false-positive rates? Bridge the gap between abstract logic scores and tangible clinical risks.

Response: We agree that connecting abstract reasoning scores to tangible clinical consequences strengthens the manuscript’s relevance. The scaphoid scenario provides a worked example. With a prior probability of 5% and a CT that is 85% sensitive and 75% specific, a positive result yields a posterior probability of approximately 15% — meaning fracture remains unlikely despite positive imaging. In a hypothetical cohort of 100 such patients, approximately 28 would receive a positive CT, of whom roughly 24 would be false positives. The 35% of surgeons who deferred to the CT over clinical assessment would therefore expose the large majority of these patients to unnecessary immobilization, additional imaging, and associated costs and anxiety — all avoidable through appropriate application of prior probability. We have added this calculation to the Discussion to explicitly bridge reasoning scores and clinical outcomes.

Manuscript change: One paragraph added to the “Base rates and prior probabilities” section of the Discussion using the scaphoid scenario to quantify the false positive burden associated with base rate neglect.

Discussion: Provide more actionable recommendations for musculoskeletal education. Given the context-dependent nature of reasoning, education should move beyond teaching formulas toward Case-Based Learning (CBL) to correct cognitive biases within specific clinical contexts.

Response: We agree that actionable educational recommendations strengthen the Conclusions. Our data suggest that because Bayesian reasoning failures are context-specific rather than uniformly distributed, educational interventions should be similarly targeted. We have added a sentence to the Conclusions explicitly recommending case-based learning and scenario-specific training as the most appropriate pedagogical approach — one that mirrors the contextual nature of the reasoning deficits observed.

Manuscript change: One sentence added to Conclusions recommending case-based learning and scenario-specific training as targeted educational interventions.

Conclusion: Emphasize the finding th

Attachment Submitted filename: Response_to_Reviewers_auresp_2.docx

# Peer review: Decision Letter 2

**DOI:** 10.1371/journal.pone.0351694.r005

**Authors:** Ismail Badr

31 May 2026

Musculoskeletal Surgeons Use Mixed Reasoning Rather Than Pure Bayesian Strategies in

Clinical Practice

PONE-D-25-55463R2

Dear Dr. Ring,

We’re pleased to inform you that your manuscript has been judged scientifically suitable for publication and will be formally accepted for publication once it meets all outstanding technical requirements.

Within one week, you’ll receive an e-mail detailing the required amendments. When these have been addressed, you’ll receive a formal acceptance letter and your manuscript will be scheduled for publication.

An invoice will be generated when your article is formally accepted. Please note, if your institution has a publishing partnership with PLOS and your article meets the relevant criteria, all or part of your publication costs will be covered. Please make sure your user information is up-to-date by logging into Editorial Manager at Editorial Manager® and clicking the ‘Update My Information' link at the top of the page. For questions related to billing, please contact billing support.

If your institution or institutions have a press office, please notify them about your upcoming paper to help maximize its impact. If they’ll be preparing press materials, please inform our press team as soon as possible -- no later than 48 hours after receiving the formal acceptance. Your manuscript will remain under strict press embargo until 2 pm Eastern Time on the date of publication. For more information, please contact onepress@plos.org.

Kind regards,

Ismail Tawfeek Abdelaziz Badr, M.D.

Academic Editor

PLOS One

**********

# Editor report: Acceptance letter

**DOI:** 10.1371/journal.pone.0351694.r006

**Authors:** Ismail Badr

PONE-D-25-55463R2

PLOS One

Dear Dr. Ring,

I'm pleased to inform you that your manuscript has been deemed suitable for publication in PLOS One. Congratulations! Your manuscript is now being handed over to our production team.

At this stage, our production department will prepare your paper for publication. This includes ensuring the following:

* All references, tables, and figures are properly cited

* All relevant supporting information is included in the manuscript submission,

* There are no issues that prevent the paper from being properly typeset

You will receive further instructions from the production team, including instructions on how to review your proof when it is ready. Please keep in mind that we are working through a large volume of accepted articles, so please give us a few days to review your paper and let you know the next and final steps.

Lastly, if your institution or institutions have a press office, please let them know about your upcoming paper now to help maximize its impact. If they'll be preparing press materials, please inform our press team within the next 48 hours. Your manuscript will remain under strict press embargo until 2 pm Eastern Time on the date of publication. For more information, please contact onepress@plos.org.

You will receive an invoice from PLOS for your publication fee after your manuscript has reached the completed accept phase. If you receive an email requesting payment before acceptance or for any other service, this may be a phishing scheme. Learn how to identify phishing emails and protect your accounts at https://explore.plos.org/phishing.

If we can help with anything else, please email us at customercare@plos.org.

Thank you for submitting your work to PLOS ONE and supporting open access.

Kind regards,

PLOS ONE Editorial Office Staff

on behalf of

Dr. Ismail Tawfeek Abdelaziz Badr

Academic Editor

PLOS One
